# Standardized in vitro analysis of the degradability of hyaluronic acid fillers by hyaluronidase

**DOI:** 10.1186/s40001-018-0334-9

**Published:** 2018-08-20

**Authors:** Bettina Alexandra Buhren, Holger Schrumpf, Edwin Bölke, Kai Kammers, Peter Arne Gerber

**Affiliations:** 1 Departments of Dermatology, Medical Faculty, Research Laboratory for Dermatology and Immunology, Heinrich-Heine-University Duesseldorf, University Hospital Duesseldorf, Duesseldorf, Germany; 2 Departments of Radiation Oncology, Medical Faculty, Research Laboratory for Dermatology and Immunology, Heinrich-Heine-University Duesseldorf, University Hospital Duesseldorf, Duesseldorf, Germany; 3 Division of Biostatistics and Bioinformatics, Department of Oncology, Sidney Kimmel Comprehensive Cancer Center, Johns Hopkins University School of Medicine, Baltimore, MD USA

## Abstract

**Background:**

Hyaluronidase is a hyaluronic acid (HA) metabolizing enzyme, which is approved as an adjuvant for infiltration anesthesia. The “off-label” use of hyaluronidase is regarded as gold standard for the management of HA-filler-associated complications. Yet, up to date there are only few studies that have systematically assessed the degradability of different HA-fillers by hyaluronidase.

**Objective:**

To analyze the interactions of HA-fillers and hyaluronidase in a time-dependent manner using a novel standardized in vitro approach.

**Methods:**

Comparable HA-fillers, Belotero Balance Lidocaine (BEL; Merz), Emervel classic (EMV; Galderma) and Juvederm Ultra 3 (JUV; Allergan), were incubated with a fluorescent dye and bovine hyaluronidase (HYAL; Hylase “Dessau”, Riemser) or control (NaCl) and monitored by time-lapse videomicroscopy. The degradation of HA-fillers was assessed as decrease in fluorescence intensity of HA-filler plus hyaluronidase vs. HA-filler plus control, quantified by computer-assisted image analysis (ImageJ).

**Results:**

Hyaluronidase showed a significant degradation of the HA-fillers BEL and EMV. Degradation was measurable at 5 h (BEL) and 7 h (EMV), respectively; significance was reached at 14 h (BEL) and 13 h (EMV). No effect of hyaluronidase was observed for JUV.

**Conclusion:**

Time-lapse microscopy enables systematically, standardized, comparative in vitro analyses of the interactions of hyaluronidase and HA-fillers.

## Background

Hyaluronic acid (hyaluronan, HA) is a non-sulfated glycosaminoglycan (GAG) and an essential part of the skin's extracellular matrix (ECM) [1, 2]. A decrease in the skin’s HA content is considered as main characteristic of skin aging [3, 4]. Today, the injection of reversible HA-based dermal fillers is regarded as gold standard for tissue augmentation, deep skin hydration or facial recontouring [5, 6]. Potential complications of filler treatments range from unaesthetic overcorrections, Tyndall effect or lower eyelid edema following tear-trough augmentation, to granulomas, infections, up to tissue necrosis or even blindness due to vascular occlusions [1]. The availability of a specific antidote, hyaluronidase, for the management of complications of filler treatments is one major reason for the preferred use of HA-based fillers over other injectable fillers, such as calcium hydroxylapatite (CHA) or poly-l-lactic acid [1, 7–9]. Since the timely infiltration of hyaluronidase may degrade HA-fillers and may rescue from more severe vascular complications, the immediate availability of hyaluronidase is regarded a necessity for every physician that injects HA [8, 10, 11]. Despite the availability of hyaluronidase, it is controversially discussed whether all HA-fillers can be degraded by hyaluronidase assimilably effective. A difference in or even resistance to “degradability” may be attributed to the concentration of HA in the filler, the degree of cross-linking, and/or its cohesive properties [12, 13]. Taking in account the potential risks associated with filler injections, the proven degradability of an HA-filler by hyaluronidase can be regarded as “safety-feature” and potential competitive edge over other manufacturers of dermal fillers.

Against this background, we here set out to systematically analyze the degradability of three commonly used HA-fillers by bovine hyaluronidase in a time dependent manner, using a novel standardized video-microscopic in vitro approach.

## Methods

**Fig. 1:**
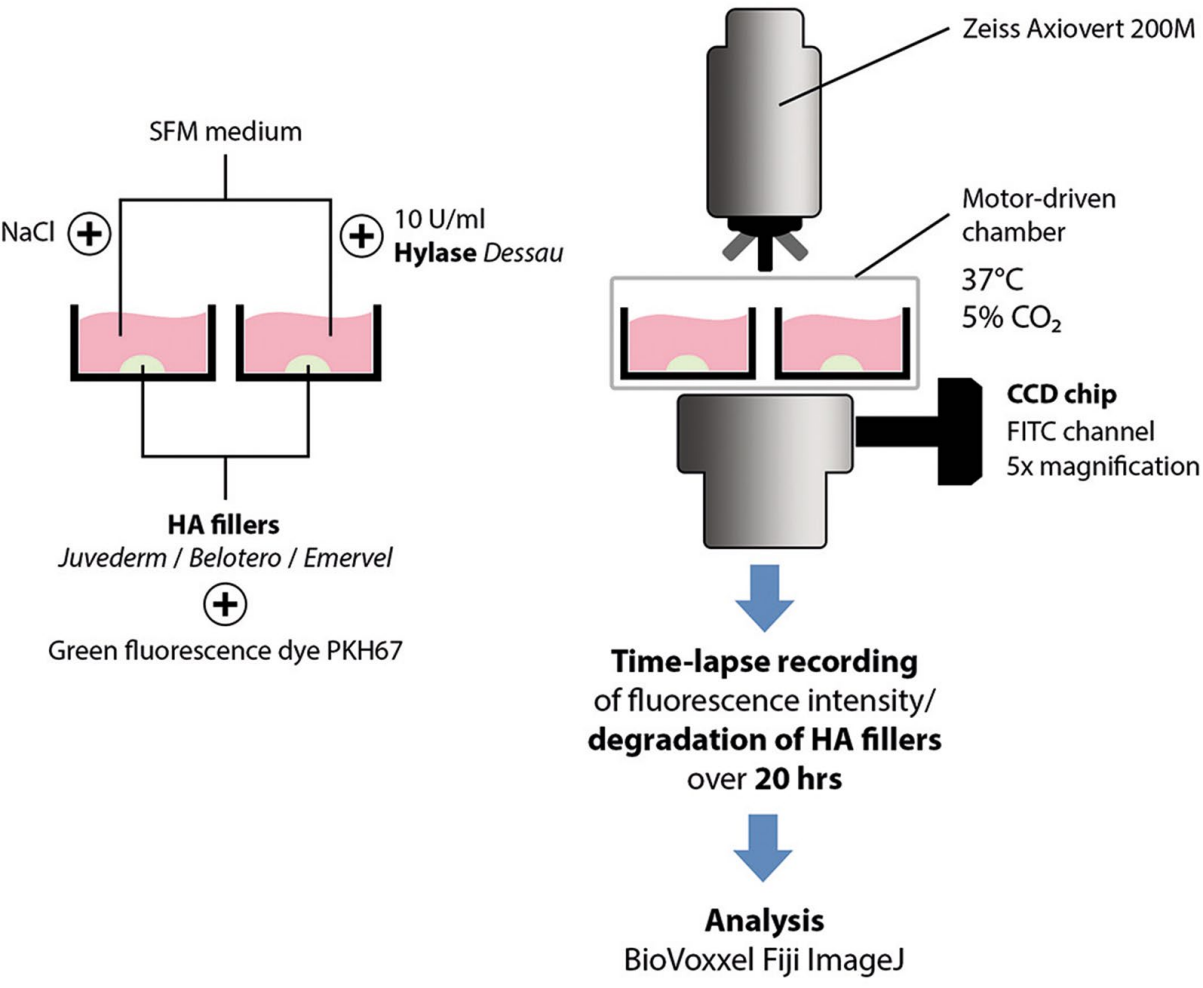
Schematic illustration of the experimental setup

For our analysis we used three comparable commercially available HA-fillers, Belotero Balance Lidocain (BEL; monophasic double cross-linked, BDDE, HA 22.5 mg/ml, with lidocaine; Merz Pharmaceuticals GmbH, Frankfurt, Germany; manufactured by ANTEIS SA, Geneva, Switzerland), Emervel classic (EMV; cross-linked, biphasic, sizing, BDDE, HA 20.0 mg/ml, lidocaine; Galderma; manufactured by Q-Med AB, Uppsala, Sweden), and Juvederm Ultra 3 (JUV; cross-linked “Hyalcross”, BDDE, HA 24.0 mg/ml, lidocaine; Pharm-Allergan, Irvine, CA, USA) [14]. First, 50 µl of each filler were mixed with 10 µl of a green fluorescent cell linker dye (PKH67, Sigma) in addition to 10 units (U/ml) of bovine testicular hyaluronidase (HYAL; Hylase “Dessau”, Riemser Pharma, Greifswald, Germany), which represents the standard hyaluronidase used in Germany, or an equal volume of NaCl and placed in 24-well plates (BD Bioscience). Next, the now fluorescent gel was placed in a larger volume of 350 µl of serum-free keratinocyte medium (SFM, Thermo Fisher Scientific) and wells were placed in a time-lapse videomicroscopy workstation (Zeiss Axiovert 200 M, Carl Zeiss Microscopy GmbH, Göttingen, Germany; 37 °C, 5% CO_2_). Depending on the integrity of the HA-filler the fluorescent dye remained either inclosed in the gel, resulting in high fluorescence intensity, or the dye was released and diluted in the surrounding medium, resulting in a decrease in fluorescence intensity. Fluorescence intensity was recorded over 20 h (5× magnification, FITC channel). Images were acquired using a high resolution monochromatic CCD chip (Zeiss). The workstation was driven by control software Axiovision 4.7 (Zeiss). The degradation of the HA-fillers was assessed as difference in fluorescence of HA-filler plus HYAL vs. HA-filler plus control (CTR; NaCl), *n* ≤ 6 per condition, quantified by computer-assisted image analysis (BioVoxxel Fiji ImageJ 1.49 m). Figure 1 shows a schematic illustration of our experimental setup.

**Fig. 2:**
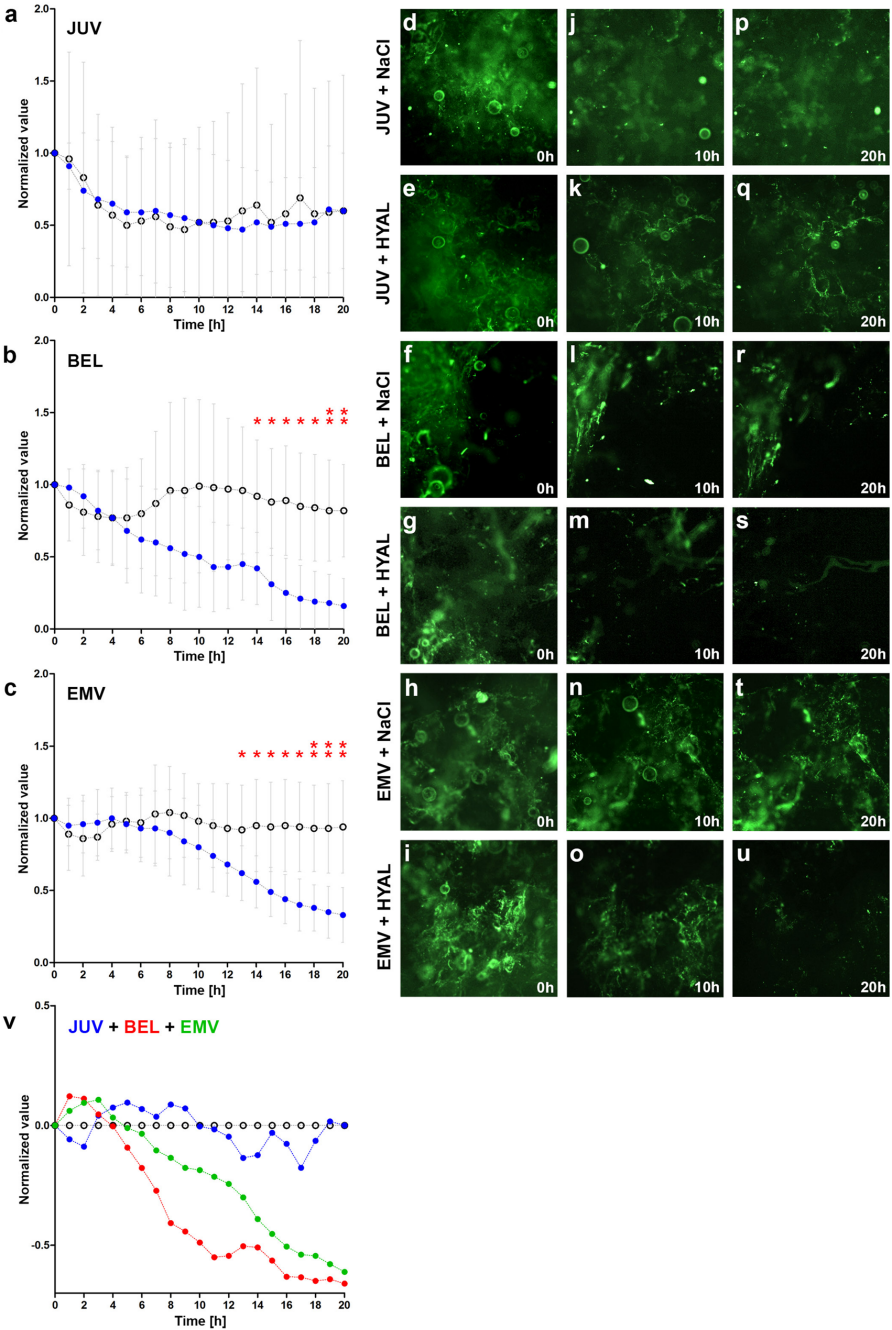
Effect of hyaluronidase on three different HA-fillers in vitro. Time-lapse videomicroscopy fluorescence analysis of the HA-fillers **a** Juvederm Ultra 3 (JUV), **b** Belotero Balance Lidocain (BEL) and **c** Emervel classic (EMV) incubated with a fluorescent dye and bovine hyaluronidase (HYAL; blue dots) or control (NaCl; black circles) in hourly intervals over 20 h. **d**–**u** Representative fluorescence images obtained at 0 h (**d**–**i**), 10 h (**j**–**o**), and 20 h (**p**–**u**) for JUV (**d**,** j**,** p** with NaCl; **e**,** k**,** q** with HYAL), BEL (**f**, **l**,** r** with NaCl; **g**, **m**,** s** with HYAL), and EMV (**h**, **n**, **t** with NaCl; **j**, **o**,** u** with HYAL). Red asterisks mark time points showing statistically significant differences of fluorescence intensity between two groups (*p < 0.05, **p < 0.01). **v** Summary of fluorescence intensities for JUV (blue dots), BEL (red dots), EMV (green dots) normalized against their respective NaCL-CTRs (zero line)

Raw fluorescence intensities were measured every 10 min and six consecutive measurements were averaged to obtain a robust value for each full hour. For easier visualization and interpretation, results were displayed on a multiplicative scale, i.e., averaged fluorescence intensities for each full hour were divided by the corresponding fluorescence intensities at time point 0 h. A value of, e.g., 0.5 in Fig. 2a–c illustrates that 50% fluorescence intensity is remaining compared to *t* = 0. At each time point, normalized fluorescence intensities between two groups were compared using a nonparametric Wilcoxon rank-sum test. Differences between two conditions with calculated *p* values smaller than 0.05 were declared statistically significant. In Fig. 2w all conditions were summarized by normalizing HYAL-treated conditions against their respective CRT-values (zero line).

## Results

All conditions (BEL + HYAL, BEL + CTR, EMV + HYAL, EMV + CTR, JUV + HYAL, JUV + CTR) showed comparable fluorescence intensities at 0 h (Fig. 2d–i). Moreover, for all conditions we documented a mild to moderate fluctuation of the overall over the course of the experiment. Significant separations of the curves for HYAL- vs. CTR-treated fillers and hence a degradation of the fillers were observed only for BEL and EMV (Fig. 2v). A degradation of BEL by HYAL (*n* = 5) was observed at 5 h and reached significance (*p* = 0.03) starting at 14 h (Fig. 2b). A degradation of EMV by HYAL (*n* = 6) was observed at 7 h and reached significance (*p* = 0.04) starting at 13 h (Fig. 2c). The strongest overall degradation was observed for BEL (Fig. 2b, s, v). No significant separation of degradation-curves and hence no effect of HYAL was observed for JUV (*n* = 4) (Fig. 2a, e, k,q).

## Discussion

Few studies have systematically assessed the degradability of HA-fillers by hyaluronidase [12–16]. In 2007 Sall et al. used a test based on the colorimetric determination of *N*-acetyl-d-glucosamine released from 11 different HA-fillers by bovine hyaluronidase [16]. Three years later Jones et al. applied an in vitro approach by analysis of the degradation products of 3 different HA-fillers treated with ovine hyaluronidase using size-exclusion chromatography [12]. In line with the results of our analysis, both studies reported the strongest resistance to degradation for the highly cross-linked 24 mg/ml HA-filler (JUV) [12, 16]. The biphasic 20 mg/ml HA-filler Restylane (equal product as Emervel classic, EMV) was reported as most sensitive [16]. In 2014 Rao et al. used a photographic approach to visually compare the interaction of human recombinant hyaluronidase (Hylenex, Halozyme Therapeutics, San Diego, CA, USA) with 4 different HA-fillers [13]. Again, the authors reported that Restylane (EMV) was degraded by hyaluronidase most effectively in a dose-dependent manner. Contrary to our results Belotero (BEL) retained its form most followed by Juvederm (JUV). Most recently, Juhász et al. conducted an in vivo human study [14]. Herein, 7 different HA-fillers, including Belotero (BEL), Restylane (EMV), and Juvederm (JUV), were injected into the back skin of 15 participants, followed by secondary injections of ovine hyaluronidase (20 or 40 units; Vitrase, Valeant Pharmaceuticals, Laval, Canada) or saline. Degradation of HA-fillers was monitored by palpation over the following 14 days. In summary, over the entire period of the observation all HA-fillers treated with hyaluronidase (20 and 40 U) showed a significant decrease in volume. In line with our analysis, Belotero (BEL) was found to be the fastest to dissolve.

Taken together, cited studies show a significant heterogeneity with regard to analyzed HA-fillers, hyaluronidases, and most notably experimental setups and techniques of analysis. As opposed to all other setups our approach is characterized by a high level of standardization, researcher-independent, computer-assisted quantification, as well as the opportunity to follow the interactions of HA-fillers and hyaluronidase over a complete time course due to time-lapse video documentation. Of note, we observed a mild to moderate fluctuation of the baseline- or CTR-fluorescence intensities for each different filler over the course of the experiment. This fluctuation is likely caused by a change in the shape of the filler, which is also evident from the representative images displayed in Fig. 2 (e.g., d, j, p; f, l, r; h, n, t). Yet, only an effective degradation of the filler and hence dilution of the dye into the medium will result in significant separations of the curves for HYAL- vs. CTR-treated fillers, which were observed for BEL and EMV. To clarify these results we plotted all values for HYAL-treated fillers against their NACL-treated controls (CTR) as displayed in Fig. 2v. In conclusion, our results are in line with previous studies, showing that hyaluronidase effectively degrades BEL and EMV [13, 14, 16].

Our results are furthermore confirmed by studies that assessed other aspects of the interaction of hyaluronidase and respective HA-fillers. In 2011 Kim et al. used a rabbit ear model to demonstrate that hyaluronidase effectively prevents skin necrosis if it is injected within 4 h after vascular occlusion of an artery using Restylane (EMV) [10]. Recently, Wang et al. used the same rabbit ear model to show that a subcutaneous injection of hyaluronidase is more effective than the intra-arterial injection. Moreover, the authors could prove that hyaluronidase effectively degrades EMV within 1 h [17]. Finally, Menzinger et al. reported that hyaluronidase effectively and dose-dependently degraded EMV in a murine model in vivo [18].

With regard to the interaction of hyaluronidase and JUV results are more inconsistent. Whereas Sall et al. and Jones et al. reported the strongest resistance to degradation against bovine or ovine hyaluronidase [12, 16], Rao et al. as well as Juhász et al. demonstrated that ovine or recombinant human hyaluronidase effectively degrades JUV [13, 14]. These controversial results could be related to differences in applied hyaluronidases (bovine, ovine, recombinant human) and respective doses, durations of incubation or experimental setups. Proposed hypotheses state that higher contents of HA as well as cross-linking-techniques have a strong effect on resistance against hyaluronidase. The strong degree of cross-linking of monophasic JUV may limit the access by the enzyme to its HA substrate, whereas the biphasic nature of EMV and its distinct particles offer a greater surface to attack [16]. In our analysis we also found that the filler with the highest content of HA (JUV, 24 mg/ml) was most resistant to degradation as compared to fillers with lower concentrations (BEL, 22.5 mg/ml; EMV, 20 mg/ml). However, we found that the monophasic BEL was comparably sensitive to degradation as the biphasic EMV.

Taken together, our study demonstrates that time-lapse videomicroscopy represents an elegant technique to assess the degradability of HA-fillers by hyaluronidase in a time-dependent manner. In this pilot-study we did not assess the effect of different doses or types of hyaluronidase. Likely higher doses of hyaluronidase may suffice to also degrade JUV, as it is evident from previous studies [13, 14]. Also higher doses of hyaluronidase will likely result in a faster degradation of HA-fillers within the first hour, like it is often observed in the in vivo or clinical situation [17]. Taking in account that hyaluronidase will not only degrade the HA-filler but also HA in the surrounding ECM it is a reasonable concern that the injection of high doses of hyaluronidase may result in a deficit of physiological HA in the treated area. Yet, the half-life and turn-over of non-stabilized, physiological HA in skin is only about 24 h, implying that equilibrium is always established within a few hours [19, 20]. In line with this hypothesis, to the best of our knowledge there are no reports on tissue deficits even after application of excessive doses of hyaluronidase, e.g., in cases of vascular occlusions following HA-filler injections. With regard to the molecular mechanisms, our results suggest that the content of HA and technique of cross-linking, but not the mono- or biphasic nature of a filler are the main factors that determine sensitivity to hyaluronidase. Future studies could extend our approach to dose–response analyses and a broader range of different HA-fillers and hyaluronidases.
